# The role of prelamin A post-translational maturation in stress response and 53BP1 recruitment

**DOI:** 10.3389/fcell.2022.1018102

**Published:** 2022-11-16

**Authors:** Cristina Capanni, Elisa Schena, Maria Letizia Di Giampietro, Alessandra Montecucco, Elisabetta Mattioli, Giovanna Lattanzi

**Affiliations:** ^1^ CNR Institute of Molecular Genetics “Luigi Luca Cavalli-Sforza”, Unit of Bologna, Bologna, Italy; ^2^ IRCCS Rizzoli Orthopedic Institute, Bologna, Italy; ^3^ CNR Institute of Molecular Genetics “Luigi Luca Cavalli-Sforza”, Pavia, Italy

**Keywords:** lamin A/C, prelamin A, DNA damage repair, laminopathies, premature ageing, Hutchinson-Gilford Progeria Syndrome (HGPS), 53BP1, oxidative stress response

## Abstract

Lamin A is a main constituent of the nuclear lamina and contributes to nuclear shaping, mechano-signaling transduction and gene regulation, thus affecting major cellular processes such as cell cycle progression and entry into senescence, cellular differentiation and stress response. The role of lamin A in stress response is particularly intriguing, yet not fully elucidated, and involves prelamin A post-translational processing. Here, we propose prelamin A as the tool that allows lamin A plasticity during oxidative stress response and permits timely 53BP1 recruitment to DNA damage foci. We show that while PCNA ubiquitination, p21 decrease and H2AX phosphorylation occur soon after stress induction in the absence of prelamin A, accumulation of non-farnesylated prelamin A follows and triggers recruitment of 53BP1 to lamin A/C complexes. Then, the following prelamin A processing steps causing transient accumulation of farnesylated prelamin A and maturation to lamin A reduce lamin A affinity for 53BP1 and favor its release and localization to DNA damage sites. Consistent with these observations, accumulation of prelamin A forms in cells under basal conditions impairs histone H2AX phosphorylation, PCNA ubiquitination and p21 degradation, thus affecting the early stages of stress response. As a whole, our results are consistent with a physiological function of prelamin A modulation during stress response aimed at timely recruitment/release of 53BP1 and other molecules required for DNA damage repair. In this context, it becomes more obvious how farnesylated prelamin A accumulation to toxic levels alters timing of DNA damage signaling and 53BP1 recruitment, thus contributing to cellular senescence and accelerated organismal aging as observed in progeroid laminopathies.

## Introduction

Lamin A is the main splicing product of *LMNA* gene and a key constituent of the nuclear lamina (Cenni et al., 2020a). The newly transcribed lamin A precursor, known as prelamin A, is 18 amino acids longer than mature lamin A and undergoes four post-translational modifications at its C-terminal CaaX box including farnesylation by the protein farnesyl transferase, double cleavage by the metalloprotease ZMPSTE24 and carboxymethylation by the methyltransferase Icmt (Cenni et al., 2014; Cenni et al., 2020a). This series of events, starting from the full-length newly translated protein known as non-farnesylated prelamin A, leads to production of full-length farnesylated prelamin A, farnesylated prelamin A lacking the last three amino acids and carboxymethylated and farnesylated prelamin A (Cenni et al., 2020a). Since processing steps are very fast under basal conditions, prelamin A forms are barely detectable in most cells and tissues. However, prelamin A levels are transiently increased upon oxidative stress (Liu et al., 2013; Cenni et al., 2014) and farnesylated prelamin A is elevated during myogenic differentiation and in differentiated muscle cells (Mattioli et al., 2011). On the other hand, *LMNA* gene mutations may affect prelamin A processing leading to toxic accumulation of different lamin A precursors, a condition that causes lipodystrophic and progeroid laminopathies (Capanni et al., 2005; Filesi et al., 2005; Cenni et al., 2014; Cenni et al., 2020a; Benedicto et al., 2022; Chen et al., 2022; Wang et al., 2022). Moreover, toxic levels of prelamin A are accumulated in tissues subjected to stress due to pathological conditions as occurs in the cardiovascular system of patients affected by chronic kidney disease (Ragnauth et al., 2010; Liu et al., 2013).

We previously demonstrated that transient reduction of prelamin A processing rate occurs in response to oxidative stress and non-farnesylated prelamin A is accumulated at the early stage of oxidative stress response (Mattioli et al., 2019). At later stages, farnesylated prelamin A becomes detectable, while only mature lamin A is present in fibroblasts after return to basal conditions (Mattioli et al., 2019). Slow-down of prelamin A processing in cells subjected to oxidative stress is in part due to downregulation of the prelamin A endoprotease ZMPSTE24 (Cenni et al., 2014; Mattioli et al., 2019), but the initial event leading to accumulation of non-farnesylated prelamin A remains unknown. However, transient prelamin A accumulation during stress response contributes to modulation of lamin A/C-HDAC2 interaction and HDAC2-dependent transcriptional regulation of p21 (Mattioli et al., 2018). In fact, lamin A/C-HDAC2 complexes are decreased few hours after the onset of DNA damage response and reformed after completion of DNA repair (Mattioli et al., 2018). Lamin A/C binding to HDAC2 favors deacetylase activity, while release of lamin A/C reduces enzyme activity and favors acetylation of histone substrates including those at the p21 gene promoter (Mattioli et al., 2018). This condition triggers upregulation of p21 during stress response. In this respect, it is worth considering that p21 decrease is necessary at the early stages of DNA damage response to allow ubiquitination of PCNA and H2A histone phosphorylation at damaged DNA, while transient p21 increase is required to avoid replication of damaged DNA sequences and in all steps to modulate DNA damage repair mechanisms (Ticli et al., 2022). However, fine tuning of p21 levels is important as unscheduled increase of p21 is a main determinant of geroconversion (Blagosklonny, 2014; Cenni et al., 2020a). Thus, the regulatory role of prelamin A in stress response appears relevant.

Another interaction involving lamin A/C during DNA damage response is the one with 53BP1, a protein recruited to DNA damage sites that in turn contributes to recruitment of other repair factors (Etourneaud et al., 2021; Paiano et al., 2021). Altered nuclear recruitment of 53BP1 has been observed in HGPS cells and ascribed to the dominant negative effect of progerin (a truncated form of farnesylated prelamin A) (Gonzalez-Suarez et al., 2011; Kreienkamp et al., 2016). On the other hand, we showed that sub-toxic levels of prelamin A contribute to 53BP1 availability in nuclei under physiological conditions and positively influence DNA repair rate in cells from long-lived individuals (Cenni et al., 2014). Moreover, we showed that non-farnesylated prelamin A accumulation occurs a few hours after oxidative stress induction, while farnesylated prelamin A is increased after 24 h and only mature lamin A is present in nuclei upon stress recovery (Mattioli et al., 2018; Mattioli et al., 2019). Here, we hypothesized that, under non-pathological conditions, modulation of lamin A/C-53BP1 binding during oxidative stress response could be linked to transient accumulation of specific prelamin A forms, i.e., non-farnesylated prelamin A or farnesylated carboxymethylated prelamin A. To test this hypothesis, we induced accumulation of those prelamin A forms, triggered an oxidative stress condition and measured the effects on 53BP1- lamin A/C interaction and 53BP1 recruitment to DNA damage foci. We also tested the effect of aberrant prelamin A accumulation at the very early stages of stress response, when only mature lamin A is detectable in nuclei. Soon after oxidative stress induction, PCNA mono-ubiquitination occurs in response to DNA damage, an event that requires reduction of p21 levels and in turn triggers gamma-H2AX histone activation (Ticli et al., 2022). Our data show that aberrant accumulation of prelamin A forms at the very early stages of stress response alters timing of PCNA ubiquitination and reduces H2AX phosphorylation. On the other hand, a few hours after stress induction, non-farnesylated prelamin A favors recruitment of 53BP1 to lamin A/C complexes, while the following prelamin A processing steps, yielding farnesylated prelamin A and mature lamin A, progressively reduce lamin A affinity for 53BP1 and favor its timely release and localization to DNA damage sites.

## Materials and methods

### Cell cultures

Human skin fibroblasts and HeLa cells were cultured at 37°C with 5% C02 in Dulbecco’s Modified Eagle’s Medium (DMEM) containing 10% heat inactivated fetal calf serum (FCS), 2 mM L-glutamine, 50 μg/ml penicillin and 50 μg/ml streptomycin. Human skin fibroblasts were from the BioLaM biobank at IGM CNR and Rizzoli Orthopedic Institute (approval prot. Gen. 0018250 del 05/09/2016). Human fibroblasts were transiently transfected with full length FLAG-tagged prelamin A (LA-WT, pCI mammalian expression vector) and mutated constructs LA-C661M, LA-L647R FuGene reagent (Roche) (Cenni et al., 2020b). The HeLa *LMNA* (*LMNA* ^−/−^) and ZMPSTE24 (ZMPSTE24^−/−^) knockout cell lines (Cenni et al., 2020b)were generated using CRISPR-Cas9 mediated genome editing technology. The guide RNA sequence which targets the first exon of the gene was: 5′- CCT​TCG​CAT​CAC​CGA​GTC​TGA​AG-3′ for *LMNA* and 5′-GGCCGAGAAGCGTATCTTCGGGG-3′for ZMSPTE24 as described before (Robijns et al., 2016). Constructs containing the Cas9 nuclease and selection markers were obtained from Addgene (#48138 and 48139) and published protocols were followed.

In human fibroblasts the accumulation of non-farnesylated prelamin A was obtained using 10 μM mevinolin (Sigma) in growth medium for 18 h while the accumulation of farnesylated and carboxymethylated-prelamin A was obtained using 50 μg/ml indinavir for 72 h. Oxidative stress was induced by the addition of H2O2 (100 μM) to the growth medium 4 h before harvesting cells (Mattioli et al., 2019). Treatment of Hela cells with H2O2 was performed as follow. After 24 h of culture, cells were treated with H2O2 (200 μM) and cells were collected at different time-points (see Figure 6) in order to follow the oxidative stress response. Lonafarnib treatment (1 μM) was performed 18 h before H2O2 administration.

### Immunofluorescence analysis

Human skin fibroblasts grown on coverslips were fixed in methanol at −20°C for 7 min. Samples were incubated with PBS containing 4% BSA to saturate non-specific binding and incubated with primary antibodies and secondary antibodies. The nuclei were then counterstained with 4,6-diamino-2-phenylindole (DAPI). The slides were mounted with an anti-fade reagent in glycerol and observed. Immunofluorescence microscopy was performed using a Nikon E600 epifluorescence microscope and a Nikon oil-immersion objective [×100 magnification, 1,3 NA (numerical aperture)]. Photographs were taken using a Nikon digital camera (DXm) and NIS-Element BR2.20 software. All images were taken at similar exposures within an experiment for each antibody. Images were processed using Adobe Photoshop (Adobe Systems).

### Proximity ligation assay

Proximity Ligation Assay (PLA) experiments was performed using kits from Sigma-Aldrich: Duolink® *in situ* Detection Reagents Orange (DUO92007). Briefly, methanol-fixed cells were saturated with saturated 4%-BSA and incubated with anti-lamin A/C (Santa Cruz sc-376248) and anti-P53BP1 (Cell Signaling 4937) primary antibodies overnight at 4°C. Thereafter, slides were incubated for 1 h at 37°C with secondary antibody-conjugated PLA probe. Ligation solution was added to each sample and slides were incubated in a humidity chamber for 30 min at 37°C. Ligation solution was removed with washing buffer and amplification solution was added. Slides were incubated in a humidity chamber for 100 min at 37°C and then washed with wash buffers. DNA was counterstained with DAPI and samples were observed by a Nikon Eclipse Ni fluorescence microscope equipped with a digital CCD camera and NIS Elements AR 4.3 software. Quantitative analysis was performed using Duolink Image Tool software (Sigma) by counting 300 nuclei per sample.

### Western blotting

For western blotting analysis cells were processed in lysis buffer containing 20 mM Tris-HCl, pH 7.5, 1% SDS, 1 mM Na3VO4, 1 mM PMSF, 5% β-mercaptoethanol and protease inhibitors. 15 μg of solubilized protein were subjected to SDS gradient gel (5%–20%) electrophoresis and transferred to nitrocellulose membrane overnight at 4°C. Incubation with primary antibodies was performed for the indicated time. Bands were revealed using the Amersham ECL detection system and analyzed with ImageJ.

### Antibodies

The antibodies employed in this study were: anti-lamin A (Abcam ab26300, diluted 1:1000 overnight at 4°C for immunofluorescence analysis) anti-prelamin A (Merck MABT858, diluted 1:500 for 1 h for Western blot analysis and 1:800 overnight at 4°C for immunofluorescence analysis) anti-lamin A/C mouse monoclonal (Santa Cruz sc-376248, diluted 1:1000 1 h for Western blot analysis); anti-PCNA mouse monoclonal (Santa Cruz sc-56, diluted 1:200 1 h for Western blot analysis); anti-P21 rabbit monoclonal (Invitrogen MA5-14949, diluted 1:2000 overnight at 4°C for Western blot analysis); anti-gamma-H2AX mouse monoclonal (Abcam 26350, diluted 1:2000 for 1 h for Western blot analysis); anti-actin goat polyclonal (SCBT I-19, diluted 1:2000 for 1 h for Western blot analysis); anti-P53BP1 (Cell Signalling 4937, diluted 1:10 overnight at 4°C for immunofluorescence analysis); anti-non-farnesylated prelamin A rabbit polyclonal (Diatheva ANT0046 diluted 1:100 overnight at 4°C for immunofluorescence analysis); anti-farnesylated prelamin A rabbit polyclonal (Diatheva ANT0045 diluted 1:10 overnight at 4°C for immunofluorescence analysis); anti-FLAG mouse monoclonal (Sigma-Aldrich M2 1:100 1 h at room temperature for immunofluorescence analysis).

## Results

### Prelamin A affects formation of 53BP1 foci under oxidative stress in human dermal fibroblasts

**FIGURE 1:**
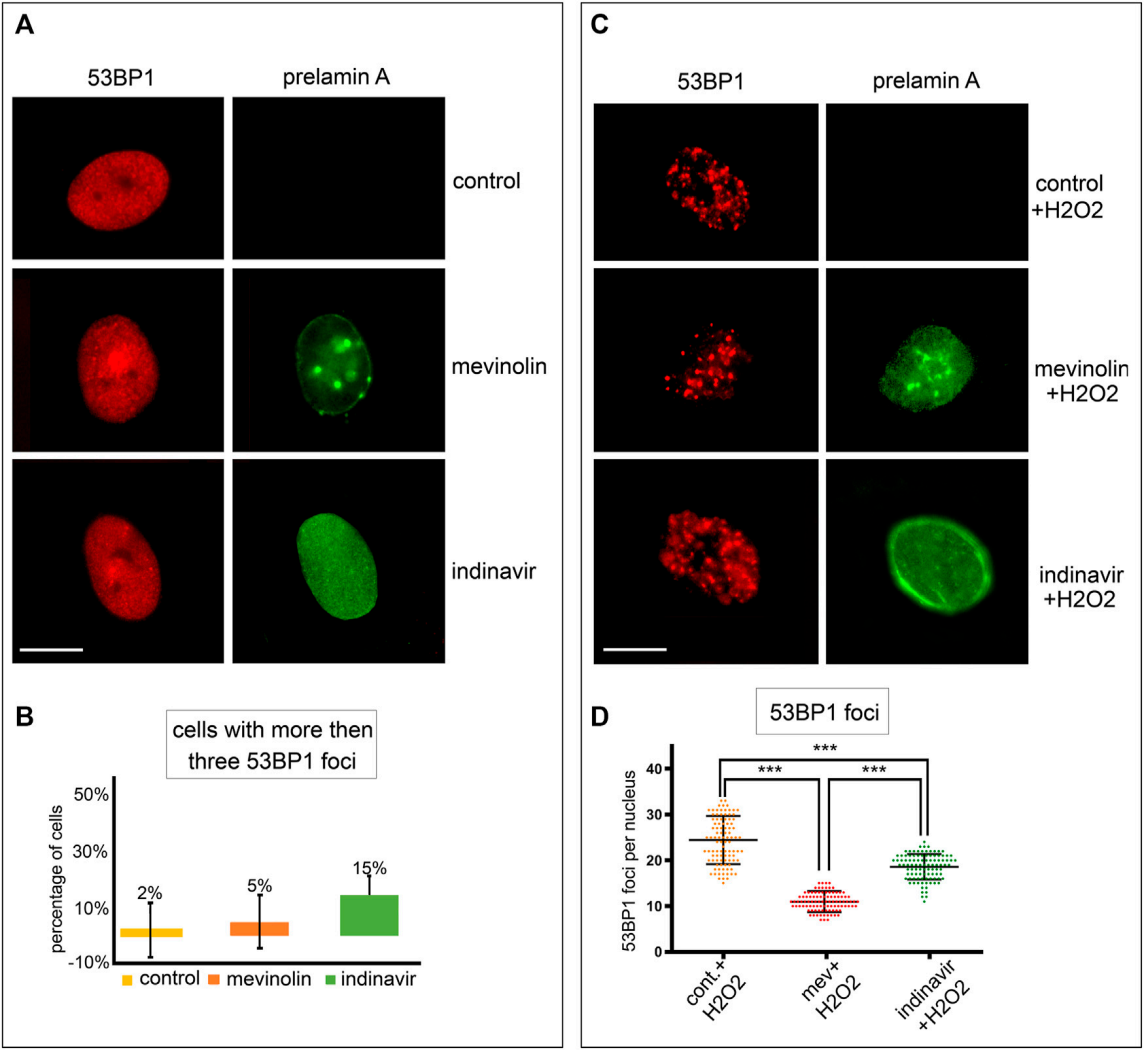
Stress-induced 53BP1 foci in human dermal fibroblasts accumulating prelamin A forms. **(A)** Immunofluorescence staining of 53BP1 and prelamin A in untreated, mevinolin- and indinavir-treated cells. 53BP1 (red), prelamin A (green). Bar, 10 μm. **(B)** Quantitative analysis of the number of fibroblasts showing more than three 53BP1 foci per nucleus. Experiments were conducted in triplicate and 200 cells per sample were counted. Data reported in the graphs are mean values +/- standard deviation. **(C)** Immunofluorescence staining of 53BP1 and prelamin A in untreated or mevinolin treated fibroblasts upon oxidative stress induction. 53BP1 (red), prelamin A (green). Bar, 10 μm. **(D)** Dot plot of the number of 53BP1 foci per nucleus in human dermal fibroblasts subjected to oxidative stress. Experiments were conducted in triplicate, data analyzed were based on an average of 100 cells. Error bars, mean ± SD. Comparison between the groups was determined by using the one-way ANOVA test. Asterisks show statistical significance (***, *p* < 0.001).

Different prelamin A forms were accumulated in human dermal fibroblasts by using prelamin A processing inhibitors. They act on well-known mechanisms, either by inhibiting farnesyl production (mevinolin, which causes accumulation of non-farnesylated prelamin A) or blocking ZMPSTE24 activity (indinavir, which causes accumulation of farnesylated and carboxymethylated prelamin A) (Coffinier et al., 2007; Dominici et al., 2009; Mattioli et al., 2019). Non-farnesylated prelamin A was undetectable in untreated fibroblasts (Figure 1A). In mevinolin-treated cells, non-farnesylated prelamin A was observed at the nuclear rim and in intranuclear foci (Figure 1A). In fibroblasts treated with indinavir, farnesylated prelamin A was observed at the nuclear rim and in the nucleoplasm (Figure 1A).

53BP1 localization was analyzed in cells that accumulated different prelamin A forms or mature lamin A under basal conditions or upon oxidative stress induction.

In unperturbed human dermal fibroblasts, 53BP1 was localized in the nucleoplasm (Figure 1A) or it was detected as one or two intensely labeled foci, while more than three 53BP1-labeled nuclear foci were observed in 2% of cells (Figures 1A,B). In this study, we assumed that cells showing three or more intranuclear 53BP1 foci were involved in the DNA damage response process.

Under basal conditions, the number of cells showing more than three 53BP1 foci in the nucleus was not significantly different between untreated and mevinolin-or indinavir-treated cells as calculated by statistical analysis, although a slight increase was observed in cells that accumulated farnesylated prelamin A upon indinavir treatment (Figures 1A,B).

In cells subjected to oxidative stress, a different scenario was observed. Four hours after H2O2 treatment, 53BP1was recruited to DNA damage sites and multiple 53BP1-labeled foci were detected in all fibroblast nuclei (Figure 1C). However, relative to cells that did not accumulate prelamin A, reduced 53BP1 recruitment in foci was observed in cells accumulating farnesylated prelamin A, while the lowest number of 53BP1 foci was detected in cells that accumulated non-farnesylated prelamin A (Figures 1C,D).

**FIGURE 2:**
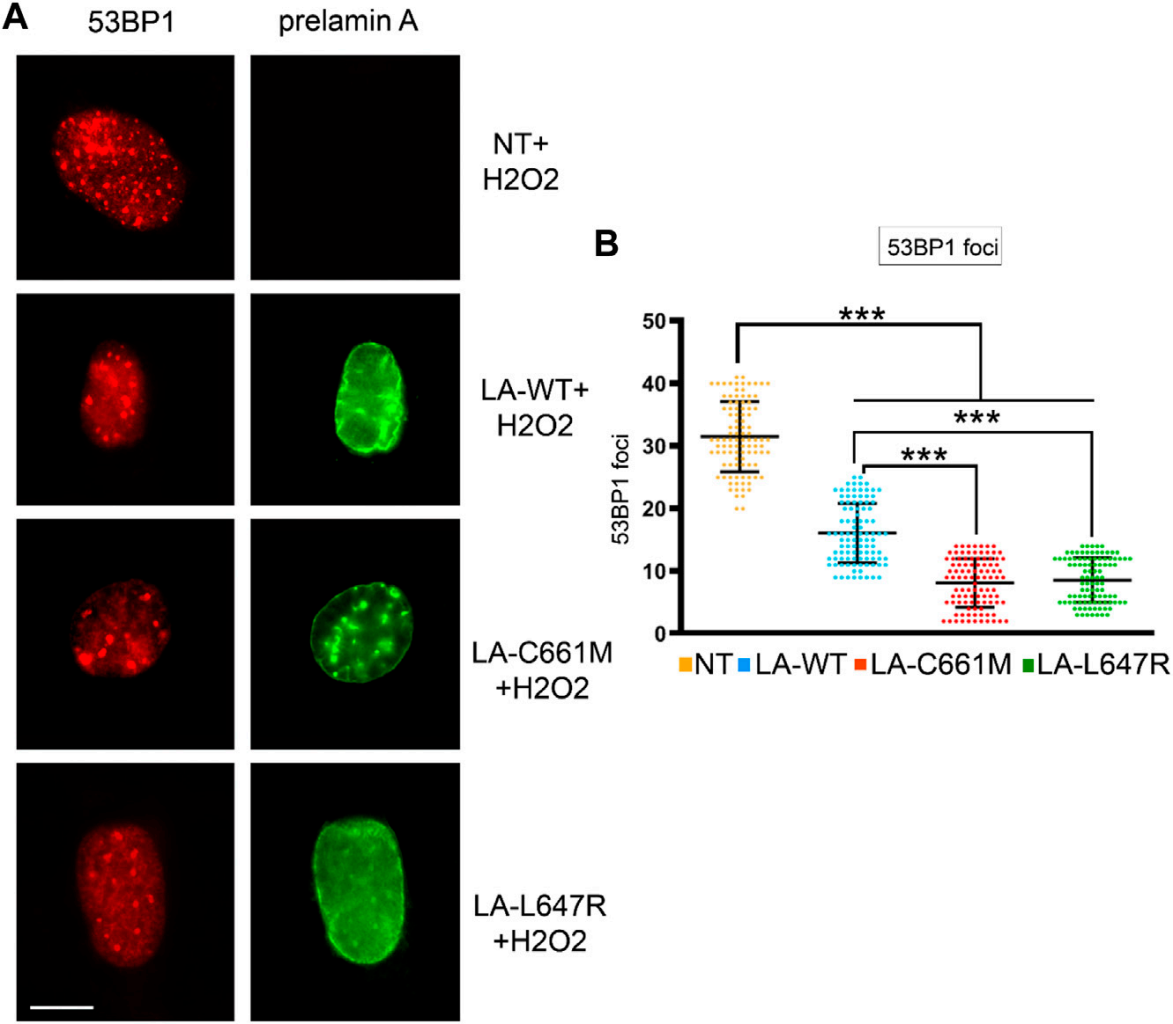
Stress-induced 53BP1 foci in human dermal fibroblasts overexpressing prelamin A mutants. **(A)** Immunofluorescence detection of 53BP1 (red) and FLAG-tagged prelamin A (green) in non-transfected fibroblasts (NT) or fibroblasts expressing wild-type prelamin A (LA-WT), non-farnesylated prelamin A (LA-C661M) or farnesylated and carboxymethylated prelamin A (LA-L647R) upon H2O2 administration. Bar, 10 μm. **(B)** Dot plot showing the number of 53BP1 foci per nucleus as detected in (A). Experiments were conducted in triplicate, data analyzed were based on an average of 100 cells. Error bars, mean ± SD. Comparison between the groups was determined by using the one-way ANOVA test. Asterisks show statistical significance (***, *p* < 0.001).

To support these observations, we overexpressed different prelamin A forms in human dermal fibroblasts and induced oxidative stress. In cells expressing LA-C661M (non-farnesylated prelamin A) a lower number of 53BP1 foci was observed with respect to cells expressing LA-WT (fully processable prelamin A) or LA-L647R (farnesylated prelamin A) (Figure 2). However, relative to mock-transfected cells, fibroblasts overexpressing any *LMNA* plasmid showed a significantly reduced number of 53BP1 foci, possibly due to accumulation of non-farnesylated prelamin A under overexpression conditions. We have observed similar behavior in other experimental settings (Capanni et al., 2012) and this observation may explain the reduced recruitment of 53BP1 to DNA damage foci even in LA-L647R (farnesylated prelamin A) with respect to LA-WT (fully processable prelamin A) (Figure 2).To avoid any bias due to overexpression conditions, the following experiments were only performed in cells that accumulated prelamin A due to mevinolin or indinavir administration.

### Accumulation of non-farnesylated prelamin A increases 53BP1 recruitment to lamin A/C complexes upon oxidative stress in human dermal fibroblasts

**FIGURE 3:**
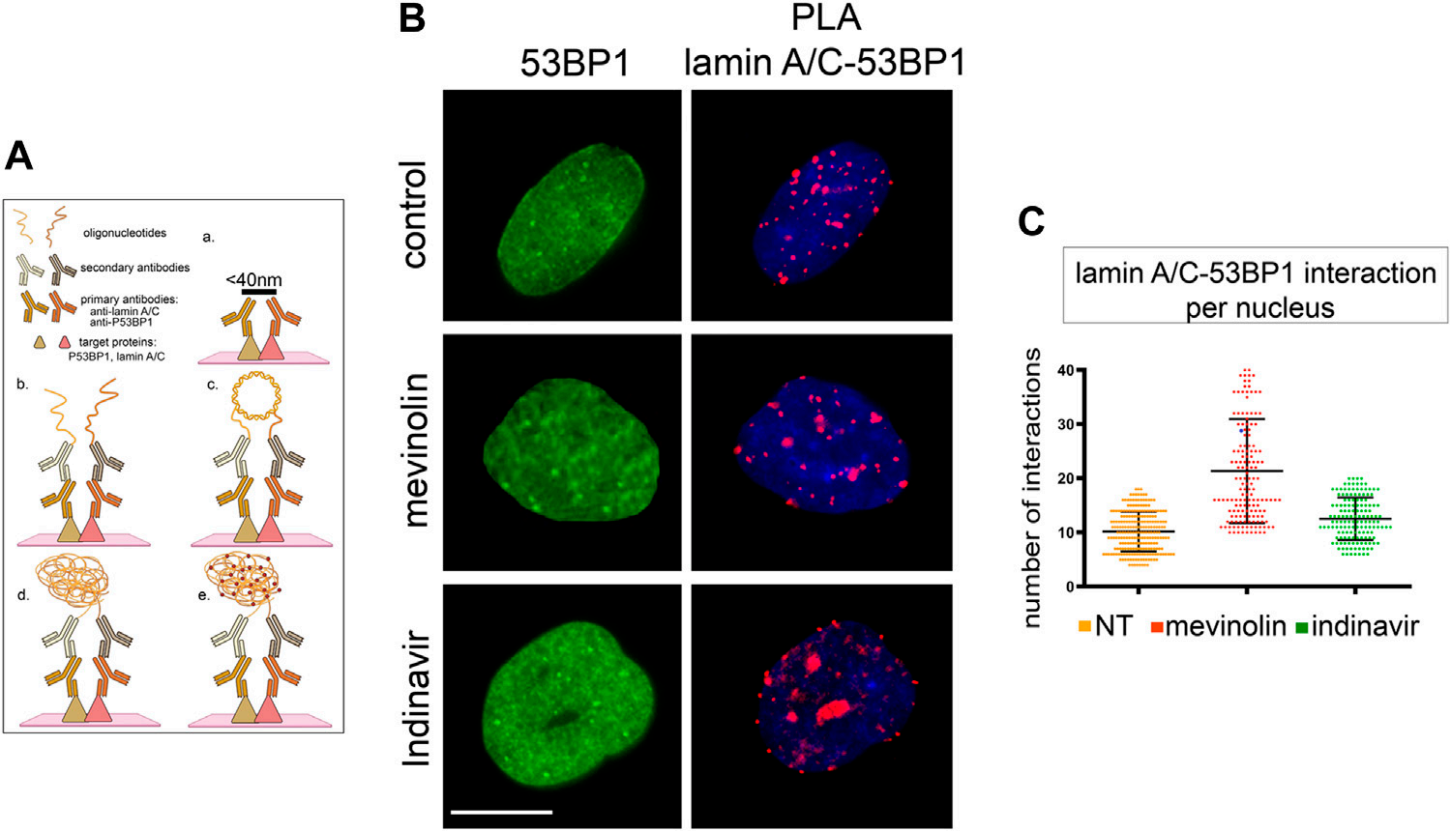
53BP1-lamin A/C binding in human dermal fibroblasts accumulating prelamin A, **(A)** Schematic representation of PLA protocol showing primary antibodies and interactors analysed in this study (created with BioRender.com). The maximum distance between interactors that allows direct binding and detection by PLA is indicated on the black bar in panel a. Binding of primary antibodies and secondary antibody-bound probes is shown in panel b. Ligase-catalysed oligonucleotide annealing is shown in c. Amplification of oligonucleotides is represented in d, incorporation of fluorescent probes is represented in e. **(B)** 53BP1 localization and PLA of lamin A/C-53BP1 interactions in untreated, mevinolin- or indinavir-treated cells. 53BP1 was detected by immunofluorescence labeling with a specific antibody (green). Interactions between lamin A/C and 53BP1 are revealed as red signals. Chromatin was counterstained with DAPI (blue). Bar, 10 μm. **(C)** Dot plot showing the number of PLA signals per nucleus as detected in (B). Experiments were conducted in triplicate, data analyzed were based on an average of 100 cells. Error bars, mean ± SD. Comparison between the groups was determined by using the one-way ANOVA test.

It has been demonstrated that 53BP1, through its Tudor domain, is able to bind lamin A/C and that this interaction is abrogated by DNA-damage (Gibbs-Seymour et al., 2015). Our hypothesis was that the accumulation of prelamin A forms could affect 53BP1 interaction with lamin A/C. Thus, we analyzed the interaction between lamin A/C and 53BP1 through Proximity Ligation Assay (PLA) (Figure3A) (Mattioli et al., 2018).

Under basal conditions, a direct interaction was detected between lamin A/C and 53BP1 (Figure3B). In mevinolin- or indinavir-treated cells, the number of interactions between lamin A/C and 53BP1 was not significantly different from that measured in untreated cells (Figure 3C).

**FIGURE 4:**
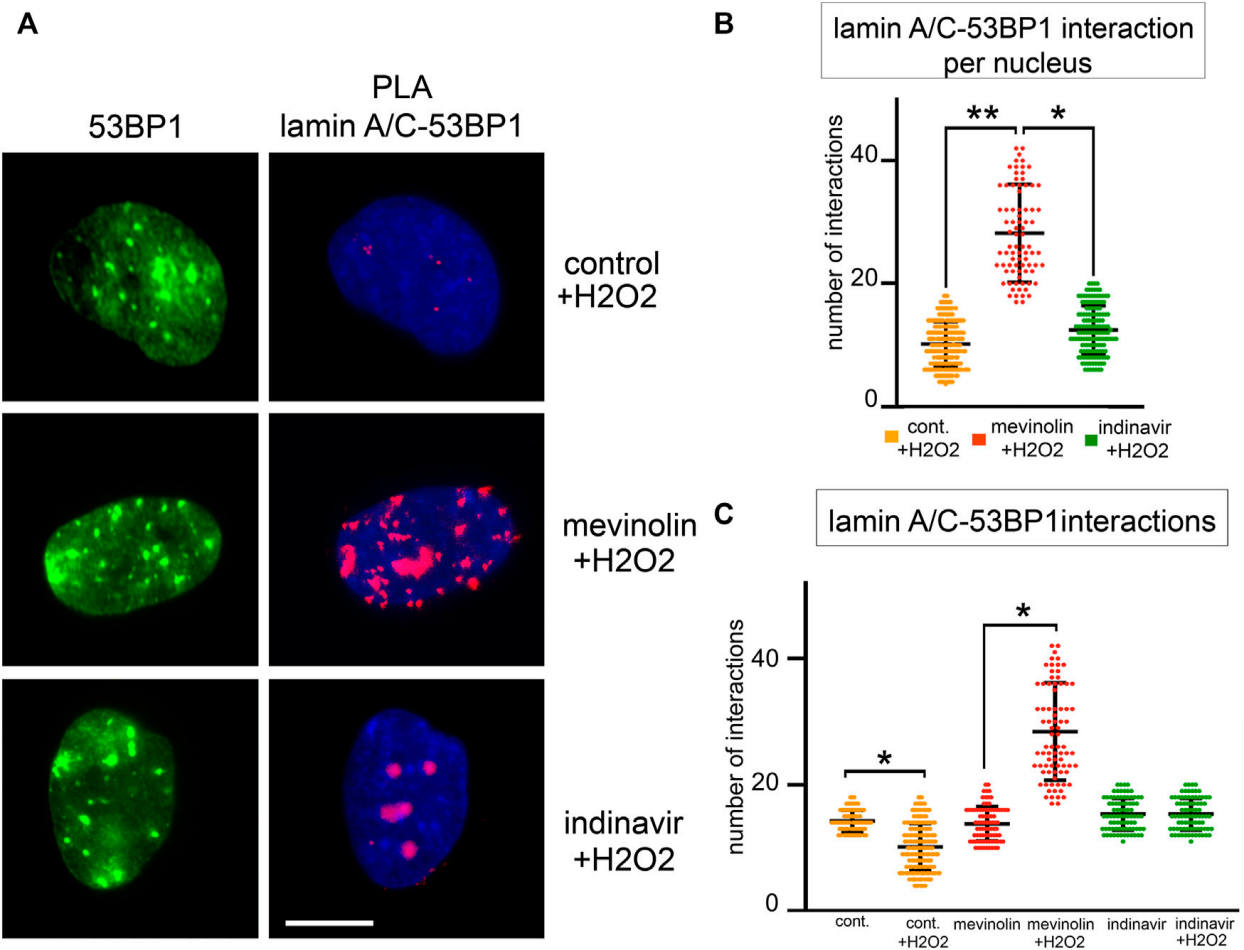
53BP1-lamin A/C binding in human dermal fibroblasts accumulating prelamin A subjected to oxidative stress. **(A)**Proximity ligation assay (PLA) showing lamin A/C-53BP1 interactions in untreated, mevinolin- and indinavir-treated fibroblasts upon oxidative stress induction. 53BP1 was detected by immunofluorescence labeling with a specific antibody (green). Interactions between lamin A/C and 53BP1 are revealed as red PLA signals. Chromatin was counterstained with DAPI (blue). Bar, 10 μm. **(B)** Dot plot showing the number of PLA signals per nucleus in fibroblasts subjected to oxidative stress. Experiments were conducted in triplicate and 100 nuclei per sample were analyzed. Comparison between the groups was determined by using the one-way ANOVA test. Error bars, mean ± SD. Asterisks show statistical significance (*, *p* < 0.05; **, *p* < 0.01). **(C)** Dot plot comparing PLA signals corresponding to lamin A/C-53BP1 interactions in fibroblasts left untreated (control) or subjected to H2O2 for 4 h (+H2O2). Untreated cells (cont.), mevinolin treated cells (mevinolin), indinavir treated cells (indinavir). Experiments were conducted in triplicate and 100 nuclei per sample were analyzed. Comparison between the groups was determined by using the one-way ANOVA test. Error bars, mean ± SD. Asterisks show statistical significance (*, *p* < 0.05; **, *p* < 0.01).

Then, we analyzed 53BP1-lamin A/C complexes and formation of 53BP1 foci upon oxidative stress induction (Figure4A–C). Four hours after H2O2 treatment, the highest number of 53BP1-lamin A/C complexes was measured in cells that accumulated non-farnesylated prelamin A, while binding signals were reduced in cells that accumulated farnesylated prelamin A and the lowest number of PLA signals was measured in cells that did not accumulate prelamin A (*p* < 0.001, Figure4B). When comparing unperturbed and H2O2-treated cells (Figure4C), the number of protein complexes was significantly reduced under stress conditions in the absence of prelamin A inhibitors (*p* < 0.001), significantly increased in mevinolin-treated fibroblasts accumulating non-farnesylated prelamin A (*p* < 0.001) and unaffected in indinavir-treated fibroblasts that accumulated farnesylated prelamin A (Figure4C). As a whole, these results were consistent with previous studies showing release of lamin A/C-53BP1 complexes upon DNA damage as a determinant of proper DNA damage response dynamics (Gonzalez-Suarez et al., 2011; Gibbs-Seymour et al., 2015) and provided evidence that prelamin A interferes with this process.

### Farnesylated prelamin A affects the early stages of stress response and 53BP1 distribution in ZMPSTE24 −/− HeLa cells

**FIGURE 5:**
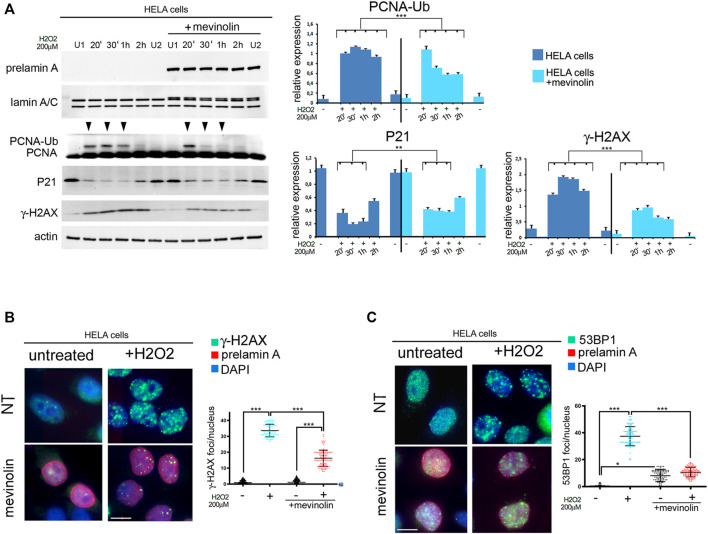
Early stage DNA damage response in HeLa cells accumulating non-farnesylated prelamin A **(A)** Western blot analysis of prelamin A (prelamin A), lamin A/C (lamin A/C), PCNA (PCNA), mono-ubiquitinated-PCNA (PCNA-Ub), P21 (P21) and gamma-H2AX (γ-H2AX) in untreated or mevinolin-treated HeLa cells (+mevinolin) subjected to oxidative stress. Actin was evaluated as a loading control. Samples were taken at various time points after H2O2 administration as indicated in each panel (20′,30′ minutes and 1, 2 h). (U1) and (U2) indicate H2O2-untreated samples collected at 30 min and 2 h, respectively. Arrowheads indicate time points in which the mono-ubiquitinated form of PCNA was detectable. Statistical analysis of mean values obtained from triplicate western blot densitometry (relative densitometry normalized to loading controls) was performed by Student’s t test. Significantly different values are indicated by asterisks: **, *p* < 0.01,***, *p* < 0.001. **(B)** Immunofluorescence analysis of gamma-H2AX (γ-H2AX, green) and prelamin A (prelamin A, red) in control (NT) or mevinolin-treated Hela cells (mevinolin) under basal conditions (untreated) and after 4 h of H2O2 administration (+H2O2). Chromatin was counterstained with DAPI (blue). Dot plot shows the number of gamma-H2AX (γ-H2AX) foci per nucleus determined in 100 nuclei. Error bars, mean ± SD. Comparison between the groups was determined by using the one-way ANOVA test. Asterisks show statistical significance (***, *p* < 0.001). **(C)** Immunofluorescence analysis of 53BP1 (green) and prelamin A (prelamin A, red) performed in control (NT) or mevinolin-treated Hela cells (mevinolin) under basal conditions (untreated) and after 4 h of H2O2 administration (+H2O2). Chromatin was counterstained with DAPI (blue). Bar, 10 μm. Dot plot shows the number of 53BP1 foci per nucleus determined in 100 nuclei. Experiments were conducted in triplicate. Error bars, mean ± SD. Comparison between the groups was determined by using the one-way ANOVA test. Asterisks show statistical significance (*, *p* < 0.05; ***, *p* < 0.001).

While the above reported data suggested a role for prelamin A in recruitment of 53BP1 during stress response, we did not observe prelamin A increase within 2 h upon stress induction (Figure 5A). Thus, we suspected that prelamin A accumulation could have a toxic effect at the very early stages of oxidative stress response. To test this hypothesis, we first assessed the effect of non-farnesylated prelamin A accumulation by measuring H2AX phosphorylation in mevinolin-treated HeLa cells soon after oxidative stress induction. In untreated and mevinolin-treated cells, phosphorylation of H2AX increased at all examined stages of oxidative stress response, including the very early time points (Figure 5A). However, lower levels of phosphorylated H2AX were detected in cells that accumulated non-farnesylated prelamin A (Figure 5A). Moreover, ubiquitination of PCNA, which is required at this stage to permit trans-lesion DNA synthesis (Cazzalini et al., 2003; Paiano et al., 2021; Ticli et al., 2022), occurred in untreated cells, while it was significantly less efficient in the presence of non-farnesylated prelamin A (Figure 5A). As p21-PCNA interaction is modulated at this stage through PCNA ubiquitination, which influences p21 degradation (Zlatanou et al., 2011), we also investigated p21 levels. In cells that did not accumulate any prelamin A form, p21 levels linearly decreased in the first stages of the stress response (20, 30 min, and 1 h) and started to increase 2 h after stress induction (Figure 5A). In the presence of non-farnesylated prelamin A, p21 decrease was observed 20 min after stress induction, while further decrease at the following time points was not observed and increased levels were detected 2 h after H2O2 administration (Figure 5A). The whole evaluation of these results supported the view that accumulation of non-farnesylated prelamin A attenuates the early DNA damage response.

Immunofluorescence analysis showed that phosphorylated H2AX at DNA damage foci was not affected by non-farnesylated prelamin A accumulation under basal conditions (Figure 5B). However, upon oxidative stress induction, while phosphorylated H2AX was detected in foci in cells that did not accumulate prelamin A, a reduced number of phosphorylated H2AX foci was formed in cells that had accumulated non-farnesylated prelamin A (Figure 5B).

53BP1 distribution was slightly affected by non-farnesylated prelamin A accumulation (mevinolin) under basal conditions (Figure 5C). However, in HeLa cells subjected to H2O2, 53BP1 foci were efficiently formed in the absence of prelamin A, while a significantly lower number of foci was detected in mevinolin-treated cells 4 h after stress induction (Figure 5C). Thus, similar effects of non-farnesylated prelamin A were determined in fibroblasts (Figure 1B) and HeLa cells (Figure 5C).

**FIGURE 6:**
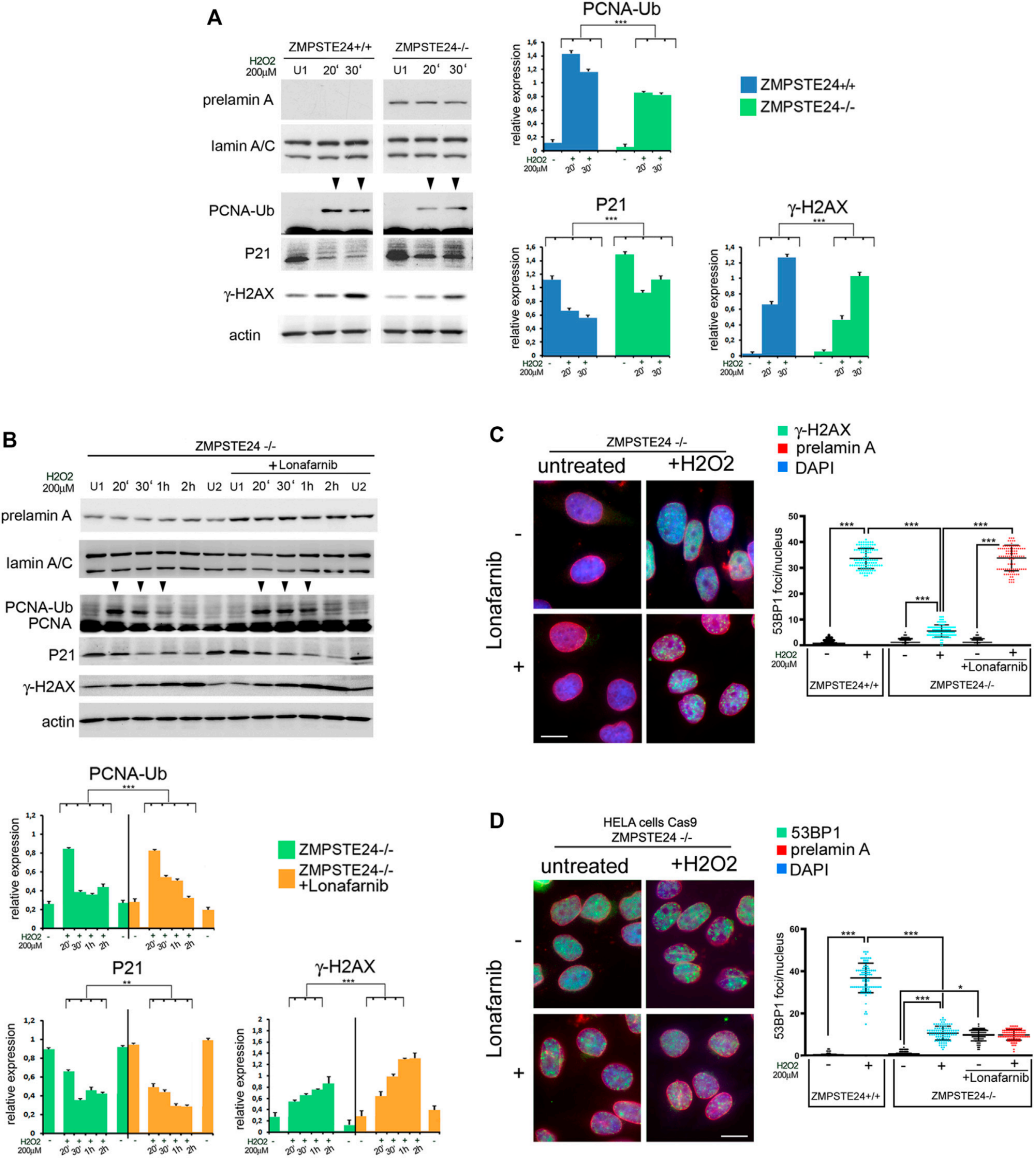
Early stage DNA damage response in ZMPSTE24^−/−^HeLa cells accumulating farnesylated prelamin A **(A)** Western blot analysis performed in ZMPSTE24^+/+^ (ZMPSTE24^+/+^) and ZMPSTE24^−/−^ HeLa cells (ZMPSTE^−/−^) subjected to oxidative stress (H2O2). Prelamin A (prelamin A), lamin A/C (lamin A/C), mono-ubiquitinated-PCNA (PCNA-Ub), P21 (P21) and gamma-H2AX (γ-H2AX) bands are shown. Actin was evaluated as a protein loading control. (U1) indicates H2O2-untreated samples collected at 30 min, while 20′ and 30′ indicates samples collected after 20 and 30 minutes after H2O2 administration respectively. Arrowheads indicate time points in which the mono-ubiquitinated form of PCNA was detectable. Statistical analysis of mean values obtained from triplicate western blot densitometry (relative densitometry normalized to loading controls) was performed by Student’s t test. Significantly different values are indicated by asterisks: *** = *p* < 0.001. **(B)** Western blot analysis performed in ZMPSTE24^−/−^ Hela cells (ZMPSTE24^−/−^) subjected to oxidative stress (H2O2) in the presence or absence of Lonafarnib (+Lonafarnib). Prelamin A (prelamin A), lamin A/C (lamin A/C), PCNA (PCNA), mono-ubiquitinated-PCNA (PCNA-Ub), P21 (P21) and gamma- H2AX (γ-H2AX) bands are shown. Actin was evaluated as a protein loading control. Samples were taken at various time points after H2O2 administration as indicated in each panel. (U1) and (U2) indicate H2O2-untreated samples collected at 30 min and 2 h, respectively. Arrowheads indicate time points in which the mono-ubiquitinated form of PCNA was detectable. Statistical analysis of mean values obtained from triplicate western blot densitometry (relative densitometry normalized to loading controls) was performed by Student’s t test. Significantly different values are indicated by asterisks: **, *p* < 0.01,***, *p* < 0.001, **(C)** Immunofluorescence analysis of gamma-H2AX (γ-H2AX, green) and prelamin A (prelamin A, red) performed in control or Lonafarnib-treated ZMPSTE24^−/−^ Hela cells under basal condition (untreated) and after 4 h of H2O2 administration (+H2O2). Chromatin was counterstained with DAPI (blue). Dot plot shows the number of gamma-H2AX foci in ZMPSTE24^+/+^ and ZMPSTE24^−/−^ Hela cells. Experiments were conducted in triplicate and 100 nuclei per sample were analyzed. Error bars, mean ± SD. Comparison between the groups was determined by using the one-way ANOVA test. Asterisks show statistical significance (***, *p* < 0.001). Bar, 10 μm, **(D)** Immunofluorescence analysis of 53BP1 (green) and prelamin A (prelamin A, red) performed in control or Lonafarnib-treated ZMPSTE24^−/−^ Hela cells under basal condition (untreated) and after 4 h of H2O2 administration (+H2O2). Chromatin was counterstained with DAPI (blue). Dot plot shows the number of gamma-H2AX foci in ZMPSTE24^+/+^ and ZMPSTE24^−/−^ Hela cells. Experiments were conducted in triplicate and 100 nuclei per sample were analyzed. Comparison between the groups was determined by using the one-way ANOVA test. Asterisks show statistical significance (*, *p* < 0.05; ***, *p* < 0.001). Bar, 10 μm.

Then, to check the effect of farnesylated prelamin A on early cellular response to oxidative stress, we took advantage of ZMPSTE24 silenced HeLa cells (ZMPSTE24^−/−^ cells) obtained by CRISPR/Cas9 gene editing. These cells accumulate farnesylated prelamin A in the absence of mature lamin A (Mattioli et al., 2019; Cenni et al., 2020b). Compared to ZMPSTE24^+/+^ cells, PCNA ubiquitination was impaired in ZMPSTE24^−/−^ cells (Figure 6A), while p21 levels were elevated under basal conditions and barely decreased upon stress induction (Figure 6A). In particular, p21 reduction was observed soon after oxidative stress induction, but fluctuation of protein levels was observed during 2 h observation (Figure 6A). Moreover, lower levels of phosphorylated H2AX were detected in ZMPSTE24^−/−^ cells upon stress induction as compared to cells that did not accumulate prelamin A (Figure 6A). The latter results showed that farnesylated prelamin A accumulation to toxic levels alters p21 modulation, ubiquitination of PCNA and H2AX phosphorylation. The whole evaluation of our data indicated that accumulation of any prelamin A form affects the very early stages of stress response and the most negative effect is observed with farnesylated prelamin A. These results were relevant to the understanding of laminopathic diseases featuring prelamin A accumulation, as the developmental disorder Restrictive Dermopathy and the progeroid syndromes Mandibuloacral Dysplasia and HGPS. As current clinical trials for progeroid laminopathies are based on inhibition of prelamin A farnesylation, we decided to investigate to which extent impairing prelamin A farnesylation in ZMPSTE24^−/−^ cells could improve early stress response. To this end, ZMPSTE24^−/−^ cells were treated with the farnesyl-transferase inhibitor Lonafarnib, which is currently used in HGPS clinical trials (Gordon et al., 2018). In Lonafarnib-treated ZMPSTE24^−/−^ cells, rescue of PCNA mono-ubiquitination was observed during the early stages of stress response (Figure 6B). Lonafarnib also increased H2AX phosphorylation levels suggesting improved recruitment of DNA damage response factors (Figure 6B).

Regarding phosphorylated H2AX distribution, we did not observe any significant difference between wild-type and ZMPSTE24^−/−^ HeLa cells under basal conditions (Figure 6C, graph). Further, lonafarnib did not affect phosphorylated H2AX under basal conditions (Figure 6C). Interestingly, oxidative stress caused a slightly (not significantly) increased number of phosphorylated H2AX foci in untreated ZMPSTE24^−/−^ HeLa cells, while foci were significantly enhanced in lonafarnib-treated cells subjected to H2O2 (Figure 6C).

As demonstrated in human dermal fibroblasts (Figures 1B, 2), formation of 53BP1 foci upon oxidative stress induction was reduced in HeLa cells that accumulated farnesylated prelamin A (Figure 6D). In fact, in ZMPSTE24^−/−^ HeLa cells subject to H2O2, a lower number of 53BP1 foci was detected relative to wild-type HeLa cells (Figure 6D, graph). However, lonafarnib treatment elicited an unexpected effect both under basal conditions and upon oxidative stress induction. In fact, the number of 53BP1 foci increased in lonafarnib-treated ZMPSTE24^−/−^ HeLa cells in the absence of any stress stimulus (Figure 6D), while H2O2 treatment did not determine any further increase of 53BP1-labeled foci (Figure 6D). Taken together, phosphorylated H2AX and 53BP1 dynamics observed in ZMPSTE24^−/−^ HeLa cells indicate that accumulation of farnesylated prelamin A uncouples DNA damage signaling (H2AX phosphorylation) from 53BP1 recruitment to damaged DNA. However, while lonafarnib restores phosphorylated H2AX recruitment, it appears ineffective towards 53BP1.

## Discussion

**FIGURE 7:**
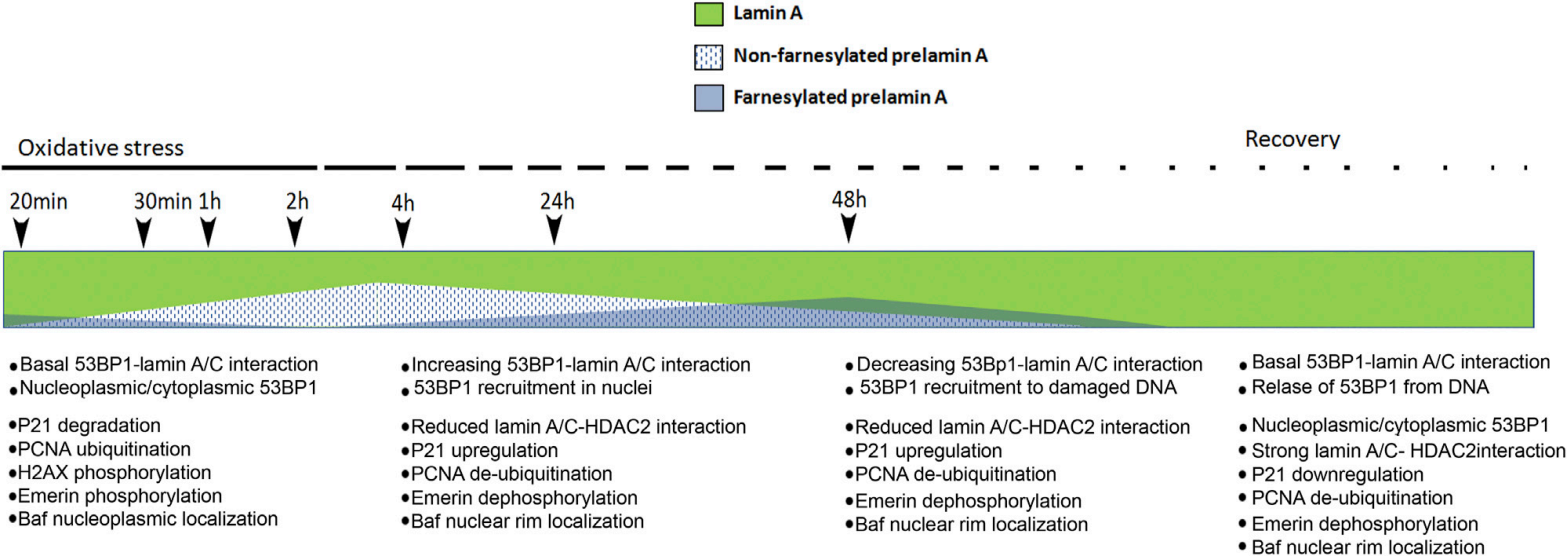
Schematic representation of our hypothesis on prelamin A role during oxidative stress response in normal dermal fibroblasts. Modulation of the relative amount of lamin A, non-farnesylated prelamin A and farnesylated prelamin A upon induction of oxidative stress is represented. Previous data showed that non-farnesylated prelamin A is accumulated in normal dermal fibroblasts 4 h after H2O2 administration and it is reduced/absent 48 h after H2O2 administration, while farnesylated prelamin A is accumulated 48 h after stress induction and it is undetectable upon stress recovery and under basal conditions (Mattioli et al., 2019). Here, we hypothesize that levels of mature lamin A (green) decrease upon slow-down of prelamin A processing, 53BP1 interactions and targeting and concomitant stress-related events are listed below the timeline. First list (20 min–1 h from stress induction, no prelamin A): at the early stage of stress response, 53BP1 interacts with lamin A/C and lamin A/C contributes to its nucleoplasmic localization (Lattanzi et al., 2007; Gibbs-Seymour et al., 2015) and targeting to a few DNA damage foci. Other events include: p21 degradation, PCNA ubiquitination, H2AX phosphorylation (this paper) and emerin phosphorylation (Cenni et al., 2020a). At this stage, emerin phosphorylation contributes to BAF release from emerin binding and BAF nucleoplasmic localization (Cenni et al., 2020b). Second list (4 h after stress induction, non farnesylated prelamin A accumulation): increased 53BP1-lamin A/C interaction elicits a low number of 53BP1 foci at DNA damage sites. In this condition, the following events occur: release of lamin A/C-HDAC2 interaction leading to p21 upregulation (Mattioli et al., 2019), PCNA de-ubiquitination (this paper), emerin de-phosphorylation (Cenni et al., 2020a). At this stage, BAF is recruited to the nuclear periphery due to emerin de-phosphorylation (Cenni et al., 2020b) and BAF-prelamin A interaction (Loi et al., 2016). Third list (48 h after H2O2 administration, farnesylated prelamin A increase followed by lamin A maturation): lamin A/C-53BP1 interaction is reduced upon prelamin A farnesylation and lamin A maturation and 53BP1 is targeted to DNA damage foci. The other events include: increase of lamin A/C-HDAC2 interaction leading to p21 down-regulation (Mattioli et al., 2019), PCNA de-ubiquitination (this paper), emerin de-phosphorylation (Cenni et al., 2020a). At this stage, BAF is recruited to the nuclear periphery (Cenni et al., 2020b). Fourth list (stress recovery, no prelamin A): lamin A/C-53BP1 interaction is maintained, leading to 53BP1 nucleoplasmic localization. The following events occur: increase of lamin A/C-HDAC2 interaction leading to p21 down-regulation (Mattioli et al., 2019), PCNA de-ubiquitination (this paper), emerin de-phosphorylation (Cenni et al., 2020a). At this stage, BAF is localized in the nuclear periphery (Cenni et al., 2020b) h, hours.

Our results bring us to the hypothesis schematically represented in Figure 7. Briefly, in cells subjected to oxidative stress, only mature lamin A is present at the very early stage of response, a condition permitting p21 decrease and PCNA ubiquitination, along with increase of H2AX phosphorylation. A few hours after stress induction, prelamin A processing is slowed-down leading to accumulation of non-farnesylated prelamin A, which elicits recruitment of 53BP1 to lamin A/C complexes and a low number of 53BP1-containing DNA damage foci. Accumulation of farnesylated prelamin A, which follows due to progression of protein maturation, causes partial release of 53BP1 from lamin A/C binding and increase of 53BP1-labeled foci. At the last stage, mature lamin A is produced, a condition associated with almost complete release of 53BP1 from lamin A/C complexes and 53BP1 targeting to DNA damage foci. Consistent with this dynamics, prelamin A accumulation affects the very early stage of stress response by impairing p21 decrease, PCNA ubiquitination and H2AX phosphorylation (Figure 7).

Prelamin A undergoes a complex post-translational processing yielding mature lamin A. This process causes formation of four different intermediates, among which, we analysed non-farnesylated prelamin A and carboxymethylated-farnesylated prelamin A (Mattioli et al., 2019; Cenni et al., 2020a). To accumulate prelamin A and test its physiological role, we decided to block its processing by using specific inhibitors and detect the endogenous proteins. In fact, any mutation in the *LMNA* sequence aimed at impairing prelamin A processing may either lead to expression of a pathogenetic prelamin A mutant (as in the case of *LMNA* L647R, which is associated with a progeroid laminopathy (Wang et al., 2016; Wang et al., 2022)) or cause toxic levels of prelamin A (Capanni et al., 2012). We can consider mevinolin and indinavir-treated cells as representative of a condition of prelamin A accumulation below the threshold level of toxicity leading to disease. On the other hand, as prelamin A is not accumulated at the very early stage of stress response, increasing its levels at that stage elicits a non-physiological condition that helps elucidating its pathogenetic pathway(s). It is worth considering that ZMPSTE24^−/−^ cells feature fully blocked prelamin A processing, thus representing a true pathological condition, while all other experimental settings used in this study recapitulate transient accumulation of different prelamin A forms in cells that also express mature lamin A, as occurs a few hours after H2O2 administration (Ragnauth et al., 2010; Cenni et al., 2014; Mattioli et al., 2019). It has been shown that prelamin A accumulation during DDR reduces lamin A/C binding to HDAC2 thus allowing chromatin relaxation due to histone H3K9 and H4K20 acetylation (Mattioli et al., 2018). Moreover, reduced lamin A/C -HDAC2 interaction during DDR affects HADC2 activity and increases the expression of p21, an HDAC2-regulated gene (Mattioli et al., 2018). As a whole, transient impairment of HDAC2-lamin A/C interaction during DDR is necessary to set chromatin and p21 in a condition allowing DNA repair (Mattioli et al., 2018).

In that context, we investigated how prelamin A accumulation interferes with lamin A/C—53BP1 binding and formation of 53BP1 foci at DNA damage sites. PLA experiments confirmed the interaction between lamin A/C and 53BP1, supporting direct binding between the two proteins (Etourneaud et al., 2021). Our results show that, under basal conditions, the presence of non-farnesylated prelamin A does not affect lamin A/C-53BP1 interaction. Consistent with this observation, formation of 53BP1 foci in nuclei is not increased in cells accumulating non-farnesylated prelamin A. This observation is relevant as it rules out major toxic effects of therapeutic treatments based on prelamin A farnesylation inhibitors as statins or FTIs, at least in dermal fibroblasts. Considering that 53BP1 foci are minimally increased under basal conditions even in cells accumulating farnesylated prelamin A, we suggest that prelamin A does not cause DNA damage *per se*, while it interferes with DNA damage repair (Cenni et al., 2014; Cenni et al., 2020a). In fact, upon induction of oxidative stress, prelamin A strongly affects 53BP1 dynamics. Non-farnesylated prelamin A is accumulated a few hours after stress induction and favours 53BP1 recruitment to lamin A/C complexes in the nucleoplasm (Lattanzi et al., 2007), possibly to collect all available protein, while the following prelamin A processing steps, ultimately eliciting mature lamin A, reduce the amount of nuclear lamina-associated 53BP1 and allow its targeting to newly damaged DNA sequences during stress response (Figure 7).

Another aspect of prelamin A-related 53BP1 dynamics is highlighted by the comparison of the number of interactions in untreated *versus* H2O2-treated cells that accumulated a specific form of prelamin A. Upon oxidative stress induction, the number of protein complexes was significantly increased in cells that accumulated non-farnesylated prelamin A, while it was reduced in cells that only expressed mature lamin A. Importantly, any oxidative stress-dependent modification (increase or decrease) in the amount of lamin A/C-53BP1 interactions relative to non-stressed cells was abolished in the presence of farnesylated prelamin A and a sort of unresponsive or locked condition was apparently established. This observation is particularly relevant to the understanding of pathological accumulation of farnesylated prelamin A. Previous studies have shown impaired 53BP1 recruitment to DNA damage foci in the presence of L647R-prelamin A (uncleavable farnesylated prelamin A (Cobb et al., 2016) and other progeria-linked prelamin A forms (Starke et al., 2013) including progerin, the truncated form of farnesylated prelamin A accumulated in HGPS (Starke et al., 2013; Kreienkamp et al., 2016). Here, we propose that persistent accumulation of farnesylated prelamin A negatively impacts two main stages of DNA damage repair. First, by affecting PCNA, P21 and H2AX modifications required at the very early stages of DDR and secondly by freezing a physiological step of farnesylated prelamin A accumulation occurring during DDR under physiological conditions. Other mechanisms and players affect 53P1 during DDR under pathological or even physiological conditions (Mayca Pozo et al., 2017). For instance, the presence of progerin has been shown to mediate cathepsin L-mediated 53BP1 degradation (Kreienkamp et al., 2016; Mayca Pozo et al., 2017). On the other hand, it has been recently demonstrated that lamin B1 forms complexes with 53BP1 and plays a key role in the regulation 53BP1 recruitment during ionizing radiation-induced DNA damage repair (Etourneaud et al., 2021). Interestingly, the authors showed that upregulation of lamin B1 alters 53BP1 recruitment to DNA damage foci by strengthening lamin B1-53BP1 complexes, while that mechanism does not involve lamin A, at least in tumour cells (Etourneaud et al., 2021). It should be interesting to investigate whether prelamin B is also accumulated in cells under stress conditions as well as the interplay between lamin B1 or prelamin A platforms recruiting 53BP1. In this study, we further show that toxic levels of farnesylated prelamin A impair PCNA mono-ubiquitination at the early stage of stress response. Mono-ubiquitination of PCNA is a key step in the activation of TLS, a DNA damage response mechanism necessary to bypass DNA lesions encountered during replication. The recruitment of polymerases specialized in DNA synthesis through damaged bases at the stalled replication fork is governed by the PCNA mono-ubiquitination, which increases the PCNA affinity for pol η (Ma et al., 2020). In this context, p21 plays a fundamental role as a negative regulator of DNA synthesis across a lesion (Soria and Gottifredi, 2010). In fact, p21 binding to PCNA impedes PCNA ubiquitination and PCNA-polymerase η interaction (Ticli et al., 2022). Here we show that the high amount of p21 elicited by unscheduled accumulation of prelamin A forms at the very early stage of DNA damage response (Mattioli et al., 2018 and, 2019) affects PCNA dynamics. This defect might add to impaired down regulation of *CDKN1A* gene upon recovery from DNA damage that occurs in cells that accumulate toxic levels of prelamin A forms as previously described in HGPS fibroblasts (Mattioli et al., 2018) and contribute to the setting of conditions that favour cellular senescence. Preventing prelamin A farnesylation improved PCNA mono-ubiquitination in ZMPSTE24 silenced cells. As progerin is a form of farnesylated prelamin A, we suggest that lonafarnib efficacy in HGPS clinical trials could in part involve improvement of PCNA processing in cells facing DNA damage. As BRCA1 supports the mono-ubiquitination of PCNA by regulating the recruitment of ubiquitinating enzymes (Tian et al., 2013), and a strong reduction in the amount of BRCA1 protein has been described in HGPS fibroblasts (Kreienkamp et al., 2016), we cannot rule out the possibility that progerin or even prelamin A accumulation could contribute to impaired PCNA ubiquitination by altering both BRCA1 and p21 levels. It is worth noting that lonafarnib treatment of ZMPSTE24^−/−^ HeLa cells was also effective in restoring phosphorylated H2AX recruitment upon oxidative stress, but did not increase the number of 53BP1-labeled foci under the same conditions. This observation was not unexpected as we had observed in fibroblasts that accumulation of non-farnesylated prelamin A (that occurs in mevinolin- or lonafarnib -treated cells) slowed-down formation of 53BP1 foci due to recruitment of 53BP1 to lamin A/C complexes. However, the apparent uncoupling between H2AX phosphorylation and 53BP1 foci formation in lonafarnib-treated ZMPSTE24^−/−^ cells could in part explain partial rescue of the HGPS cellular phenotype upon lonafarnib administration.

Our study here reported has been performed in dermal fibroblasts and epithelial cells. This represents a limitation of the study, as different prelamin A-related dynamics might occur in different cell types, also depending on different nuclear envelope interactors of lamin A/C (Tingey et al., 2019; Czapiewski et al., 2022). A possibility exists that prelamin A in either form, governs timing of DNA damage repair thus fitting repair dynamics to pre-existing cellular conditions. This could be the case of myotubes and muscle cells, where farnesylated prelamin A is detectable even in the absence of stress conditions (Mattioli et al., 2011). Moreover, provided that an analogous mechanism of 53BP1 modulation is mediated by lamin B1 in bone or epithelial tumour cells (Etourneaud et al., 2021), it should be interesting to investigate whether different lamin platforms anchor 53BP1 in transformed cells.

## Conclusion

The whole evaluation of different players involved in DDR will allow us to discriminate whether prelamin A accumulation is a trigger of DNA damage or an activator of DNA repair factors under different stress conditions. In our opinion, both hypotheses can be true, as we see that modulation of prelamin A post-translational processing rate contributes to proper timing of DNA damage repair. As a consequence, altered prelamin A modulation may impair DNA damage repair and increase the amount of unrepaired DNA.

As a whole, our data show that prelamin A does not induce *per se* DNA-damage when transiently accumulated at low levels in the absence of stress stimuli. However, soon after stress induction, prelamin A accumulation appears to impair proper DNA damage response due to inhibition of PCNA ubiquitination. On the other hand, transient increase of prelamin A levels at the following stages is crucial to modulate 53BP1 targeting to DNA-damage sites. We can assess that non-farnesylated prelamin A favours recruitment of 53BP1 to lamin A/C-containing complexes, while prelamin A processing allows timely 53BP1release. Based on previous and present observations, we suggest that the increased lamin A/C-53BP1 interaction occurring in the presence of non-farnesylated prelamin A helps collecting all available 53BP1 from inside and outside the nuclei. Alternatively, recruitment of 53BP1 by non-farnesylated prelamin A could establish a priority in damaged DNA to be repaired. It will be interesting to establish to which damaged sequences is 53BP1targeted in the presence of non-farnesylated prelamin A, if those sequences are within lamina-associated chromatin domains (LADs) (Robson et al., 2017; Bellanger et al., 2022; Madsen-Osterbye et al., 2022), correspond to the most recently damaged DNA or are they selected by any lamin A/nuclear envelope-related tissue-specific mechanism (Mattioli et al., 2011; Czapiewski et al., 2022).

In this context, we propose that senescent cells are accumulated in patients affected by progeroid laminopathies due to unscheduled or impaired activation of prelamin A-regulated DNA damage repair mechanisms under oxidative stress conditions. Thus, clearly the genome defect is a cause of the pathology. However, we suggest that other prelamin A-dependent events not related to DNA damage response, as for instance remodeling of specific chromatin domains (Bellanger et al., 2022) or recruitment of chromatin binding proteins as BAF, LAP2alpha or HDAC2 (Lattanzi et al., 2007; Mattioli et al., 2008; Mattioli et al., 2011; Camozzi et al., 2014; Loi et al., 2016; Mattioli et al., 2018) contribute to the onset of organismal ageing when one or more prelamin A forms are stabilized. As a whole, we believe that un-processable prelamin A forms cannot accomplish their main role of sensors of “environmental changes” and timely activators of specific stress responses (Cenni et al., 2020a) as any of these functions relies on modulation of prelamin A maturation rate or establishment of transient interactions at the prelamin A-specific C-terminal domain. This has been shown for p21, HDAC2, LAP2alpha, Oct-1, BAF in cells subjected to oxidative stress and all these proteins impact on genome functional organization (Mattioli et al., 2008; Cenni et al., 2014; Mattioli et al., 2018; Mattioli et al., 2019; Cenni et al., 2020b). On the other hand, it is too complex of an issue to be able to completely rule out that other functions of the prelamin A are indirectly responsible for the increases in DNA damage. For instance, a direct consequence of the accumulation of progerin or toxic levels of wild-type prelamin A is a dramatic remodeling of the whole nuclear envelope (Columbaro et al., 2005; Columbaro et al., 2010; Pellegrini et al., 2015; Squarzoni et al., 2021) with deleterious effects on cytoskeleton interactions (Balmus et al., 2018) and chromatin spatial organization leading to formation of damaged cytoplasmic DNA (Graziano et al., 2018; Kreienkamp et al., 2018). This in turn may activate inflammatory responses driven by the NF-kB and Jak/STAT pathway (Liu et al., 2019; Squarzoni et al., 2021) and cause chromatin reorganization as a response to inflammation. From this point of view, the pathology is a cause of genome defects. As all these considerations can be considered acceptable, it is not surprising that a vicious circle set up in progeroid laminopathies leads to worsening of cellular and organism phenotype in a very short time-frame. Our results and the above reported considerations may contribute to unravelling fine tuning of DNA damage repair mechanisms and chromatin dynamics, which occur under physiological conditions and avoid genome instability and cancer on one side and cellular and organismal aging on the other side.

## Data Availability

The raw data supporting the conclusion of this article will be made available by the authors, without undue reservation.
